# A Fault-Diagnosis Method for Railway Turnout Systems Based on Improved Autoencoder and Data Augmentation

**DOI:** 10.3390/s22239438

**Published:** 2022-12-02

**Authors:** Mengyang Li, Xinhong Hei, Wenjiang Ji, Lei Zhu, Yichuan Wang, Yuan Qiu

**Affiliations:** School of Computer Science and Engineering, Xi’an University of Technology, Xi’an 710048, China

**Keywords:** fault diagnosis, railway turnout system, unbalanced datasets, improved autoencoder, data augmentation

## Abstract

In recent years, with the rapid increase in coverage and lines, security maintenance has become one of the top concerns with regard to railway transportation in China. As the key transportation infrastructure, the railway turnout system (RTS) plays a vital role in transportation, which will cause incalculable losses when accidents occur. The traditional fault-diagnosis and maintenance methods of the RTS are no longer applicable to the growing amount of data, so intelligent fault diagnosis has become a research hotspot. However, the key challenge of RTS intelligent fault diagnosis is to effectively extract the deep features in the signal and accurately identify failure modes in the face of unbalanced datasets. To solve the above two problems, this paper focuses on unbalanced data and proposes a fault-diagnosis method based on an improved autoencoder and data augmentation, which realizes deep feature extraction and fault identification of unbalanced data. An improved autoencoder is proposed to smooth the noise and extract the deep features to overcome the noise fluctuation caused by the physical characteristics of the data. Then, synthetic minority oversampling technology (SMOTE) is utilized to effectively expand the fault types and solve the problem of unbalanced datasets. Furthermore, the health state is identified by the Softmax regression model that is trained with the balanced characteristics data, which improves the diagnosis precision and generalization ability. Finally, different experiments are conducted on a real dataset based on a railway station in China, and the average diagnostic accuracy reaches 99.13% superior to other methods, which indicates the effectiveness and feasibility of the proposed method.

## 1. Introduction

The railway, as part of the national transportation infrastructure, undertakes a large number of transportation tasks for freight and passengers, incurring incalculable losses when accidents occur. In recent years, with the rapid increase in coverage and lines, fault diagnosis and security maintenance for railways gradually become one of the top concerns in China [1,2,3]. The intelligence fault diagnosis of key railway equipment, such as turnout [4], rolling bearings [5], gearboxes [6], and brakes [7], etc., has been achieving wide application due to the rapid development of artificial intelligence techniques, sensor technology, and computer technology. The railway turnout system (RTS) is one of the significant structures of the railway infrastructure, which is essential to control the running direction of the train and ensure running safety. The intelligent fault diagnosis of RTS has become a hotspot, which is challenged in two aspects: feature characterization and data imbalance.

The constitution of RTS consists of the onsite part and the indoor power monitoring part [8]. The turnout of the onsite part mainly relies on the power provided by the switch to complete the action. During the turnout transformation, the switch-machine motors load the electronic current, and power signals are monitored by the microcomputer monitoring system (MMS) in the indoor power monitoring part, which is displayed as a current or power curve. However, the turnout is prone to failure due to its complex electromechanical structure, long-term exposure to the outdoor environment, and frequent pulling of trains. The existing daily fault diagnosis and security maintenance mainly rely on monitoring the turnout power or current curves in real time from MMS. Then, the turnout state will be given by a fixed threshold judgment and engineering analysis based on the displayed curves, which are not only low in diagnosis efficiency but which also hardly review the health of RTS. Consequently, false or missing alarms happen frequently in traditional maintenance approaches, and the task of RTS fault diagnosis in daily maintenance should be more accurate, efficient, and intelligent, and conducted in real-time.

At present, various articles in the literature have put forward the application of artificial intelligence technology to the fault diagnosis of RTS, and the related methods can be summarized as the threshold-based method [9,10], machine learning (ML) based [11,12,13,14,15,16,17,18,19,20,21,22], or deep learning (DL) based [23,24,25,26]. Huang et al. [9] proposed a fault-detection method by using dynamic time warping based on the turnout current curve, which can detect faults by comparing the distance from the template curve. However, this kind of diagnosis method excessively relies on the fixed threshold settings from expert experience and is deficient in accuracy and generalization. To avoid these drawbacks, various research based on ML aimed to relatively reduced participation of manual settings, and it has been proven to be efficient and accurate in the field of fault diagnosis. Unsupervised learning and supervised learning are two types of ML [14,15]. Based on the feature engineering, several classification unsupervised methods, such as fuzzy cognitive map [16], support vector machine (SVM) [17], backpropagation [18], and ensemble classifier [19], have been presented in order to find the best combination features/classifiers to make intelligent decisions regarding the presence of RTS. In particular, SVM has been widely applied to evaluate condition monitoring as a supervised method. Guo et al. [20] proposed a hybrid feature-extraction method, which combines time and frequency domain; then, the SVM is further used for classification purposes. Li et al. [16] constructed a new type of ZD6 turnout system fault-diagnosis model by using a wavelet transform feature extraction and fuzzy cognitive map classification. In [22], Lee et al. employed SVM for early detection and classification of audio data of railway point devices, and the experiment results show that it has a good effect.

Although ML-based fault diagnosis has achieved some good results, it relies too much on feature engineering based on signal processing technology, which cannot successfully get deep feature information from the signals. DL-based fault-diagnosis methods have been proposed and have recently gained considerable attention with the development of sensor technology. Specifically, Zhang et al. [23] proposed an intelligent fault-diagnosis method for high speed, in which the turnout current signals in time domain are converted to the 2D grayscale images, and then the grayscale images are fed into the CNN for classification. Similarly, Huang [24] input the acquired current pictures directly to CNN for classification. Kou et al. [25] applied CNN to automatic feature learning and classification for rotating devices fault diagnosis on a high-speed train bogie. For the historical field data collected from a real high-speed railway, Guo et al. [26] proposed a detection method based on stacked autoencoders (SAE), which is pretrained by layers and followed by a fine-tuning phase. Consequently, feature extraction and fault detection were integrated into one step.

Fault-diagnosis technology of the RTS generally includes several steps: (i) data preprocessing, (ii) feature extraction, (iii) feature selection, (iv) data augmentation, and (V) classification. Similar to other industrial systems, signal acquisition is limited by diverse MMSs, different railway bureaus, and terrible environments. Generally, the collected signals have characteristics such as inconsistent length and noise, Thus, it is of particular significance for data preprocessing of signals before fault diagnosis. Articles in the literature [27,28] adopt normalization for raw data and feature data to ensure the consistency of length. A wavelet packet is also used for de-noising rolling bearing data [29]. Yang et al. [30] proposed an improved sparse autoencoder and multilevel denoising strategy (MDS-ISAE) for diagnosing the early multiple intermittent faults of gearboxes and rolling bearings, respectively. Relatively speaking, the environment where the turnout is located is worse, and the collected power signal data is inevitably interfered with by noise. An excellent fault diagnosis depends on high-quality data samples, leading to the necessity of data preprocessing.

Feature extraction and feature selection are the main processes in artificial feature extraction [22]. Feature extraction obtains new features containing information reflecting the states of machines with obvious physical or statistical significance by extracting features through various mapping functions. Traditional feature-extraction methods include extraction from the time-domain, the frequency domain [31], the time–frequency domain, and data-mining methods (like principal component analysis (PCA) and linear discriminant analysis (LDA) [32]). To weaken the redundant information contained in feature extraction, reduce feature dimensions, and save computational costs, some feature-selection methods (e.g., filter, wrapper, and embedding) are used to obtain sensitive information about the health status of machines from the collected ones [33]. However, most traditional feature-extraction and feature-selection methods are limitations, which heavily rely on domain knowledge, which leads to the challenge of uncovering the nonlinear correlations between the latent features in high-dimensional data.

To overcome the aforementioned weaknesses, developing a method by which to adaptively learn the features from raw signalizing advanced artificial intelligent techniques would be necessary, instead of extracting and selecting features manually. As a successful DL technology, the autoencoder (AE) with the potential of latent feature extraction has been applied [8,12,34,35] widely. Compared with traditional feature-extraction methods, AE extracts the deep feature information in the signal more effectively and depends less on prior knowledge or human labor. Li et al. [35] employed a locally connected autoencoder to automatically capture high-order features. In this work, the current signals are segmented and blended to enhance the temporal characteristic. AE-based fault diagnoses in industrial systems are proposed [30], revealing that real artificial intelligence can be achieved without manual participation. Some scholars apply unsupervised learning to the RTS fault diagnosis. To predict the faults of RTS caused by insufficient lubrication, Zhang et al. [8] utilized the unsupervised learning ability of an improved sparse AE to select the best feature collection. An unsupervised fault-detection algorithm based on deep autoencoders (DAE), which was used to extract high-level features, was proposed to improve the overall fault-detection performance in the literature [12]. Specifically, compared with traditional manual feature-extraction approaches, AE is more suitable for the feature extraction of high-dimensional data and can excavate more latent features, and this paper focuses on the fault-diagnosis method that combines denoising and feature extraction.

Currently, as far as RTS fault diagnosis is concerned, numerous studies assume that the training datasets are balanced, and they have achieved excellent results. However, during the railway daily operation, the RTS signals collected by MMS always result to unbalanced dataset. Data enhancement is applied in the field of fault diagnosis by some scholars. A variable-scale resampling strategy, which employed the large shift and less overlap resampling to obtain the sample sets, is designed to solve the problem of fault diagnosis from imbalanced data [36]. The resampling strategy is more suitable for data without obvious phases, such as vibration signal, rather than the power of RTS. Articles in the literature [37] developed a novel method based on an improved generative adversarial network (GAN) for imbalanced fault diagnosis of a planetary gearbox. GAN is not stable enough for data enhancement of one-dimensional signals. Before category identification, the synthetic minority oversampling technique (SMOTE) was used to balance datasets [38]. In contrast, SMOTE has a more stable data enhancement effect through its simple linear interpolation.

The traditional turnout fault diagnosis have achieved some good results, but they still have trouble with feature characterization and data imbalance. To solve the above problems, this paper aims to the turnout fault diagnosis of RTS and proposes a new fault-diagnosis method for RTS based on an improved autoencoder and data augmentation method. The contributions of this paper are summarized as follows.

1. We design an improved autoencoder that combines smooth denoising and feature extraction to achieve deep feature extraction of turnout power curves. We manually construct and select high-quality datasets for training smooth denoising autoencoders (SDAE), the purpose of which is to realize the smooth denoising function of the autoencoder on the original dataset and reduce the noise interference problem caused specifically by physics. Then, smooth denoising and feature extraction autoencoder (SD-FAE), which obtains latent features by unsupervised learning, is constituted by stacking two fully connected layers after SDAE. Compared with traditional manual feature-extraction approaches, it is more suitable for feature extraction of high-dimensional data and can excavate more latent features of RTS.
2. In order to solve the imbalanced data problem of RTS, after unsupervised feature extraction based on SD-FAE, each type of fault feature dataset is expanded to the same number as the healthy dataset by SMOTE. In the experiment, we further verified the effectiveness and correctness of data enhancement in RTS fault diagnosis by LDA 3D visualization.
3. Based on a real dataset marked by experts, the performance of the model proposed by us in accuracy, precision, recall and F1- score are all higher than that in the existing literature after testing the method suggested in this research with a real and manually marked dataset, and the average diagnostic accuracy reaches 99.13%.

The organization of this paper consists of four sections after the introduction. In Section 2, the basic structure and background knowledge of RTS and its imbalance dataset that is a collection of MMS is briefly introduced. The framework and the proposed approach for an autoencoder that combines smoothing and feature extraction, data augmentation, and a classification algorithm are illustrated in Section 3. The experimental parameter settings, evaluation indicators, and findings are displayed in Section 4. Finally, Section 5 concludes this paper.

## 2. Railway Turnout System

The railway turnout system is one of the most critical pieces of equipment in railway infrastructure and plays a vital role in railway operation [39]. The turnout system, which controls the running direction of the train and realizes the transfer of the train from one track to another, is run by motors. To achieve the safe operation of railways, MMS has been widely introduced to timely monitor switch states in China [12,32]. Typically, MMS gathers power and current curves for turnout operation, allowing maintenance personnel to assess the condition of switches and provide diagnosis based on their knowledge. However, a lack of experience can lead to missing or false alarms, both of which pose serious security risks. Furthermore, the number of turnout action curves is relatively large, and many financial and human resources are involved in such work. The RTS functions on the basis that the action power curve reflects the operation and deterioration of the mechanical parts of the RTS, whereas the current curve indicates the state of operation of the control circuit of the system [8]. As a result, the study object for the RTS problem diagnosis is chosen to be the power curve gathered by the MMS.

**Figure 1:**
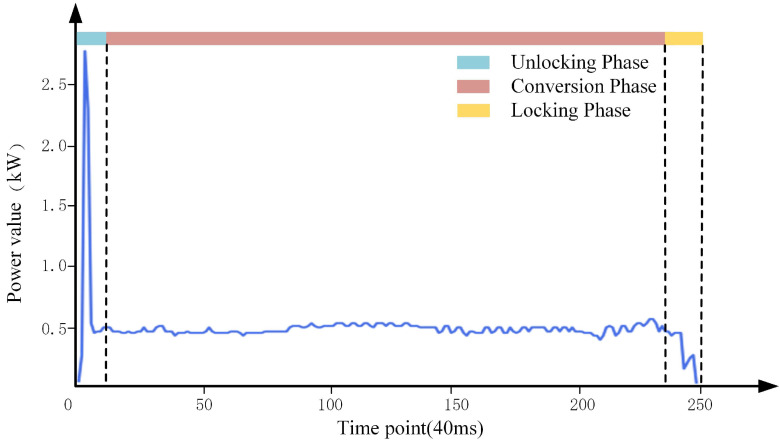
Turnout health power curve.

Driven by electric energy, the switch machine supports turnout switching, which will generate a current or power curve. Figure 1 shows the healthy power curve of the ZD6 switch machine, which is generally divided into three stages: unlocking, conversion, and locking. The vertical ordinate in the figure represents the power value (kW), the abscissa represents the time series during the power value of the turnout, and the interval time is 40 (ms). The unlocking phase is the process of unlocking the turnout; the motor drives the turnout to start switching in a short time, and then the power curve trend is to rise to a maximum value first and then decrease. What follows is the conversion phase; this stage is the process from the starting position to the target position of the switch rail. The turnout transforms smoothly, and the power curve is basically smooth. The locking phase is the last phase. The switch rail reaches the target position, and the turnout conversion is completed. We have the motor finish energization, and then the power curve drops sharply and remains at zero. Normally, the trend of the power curve is the same as in the above three stages. Once the fault occurs, the curve trend will be changed.

**Figure 2:**
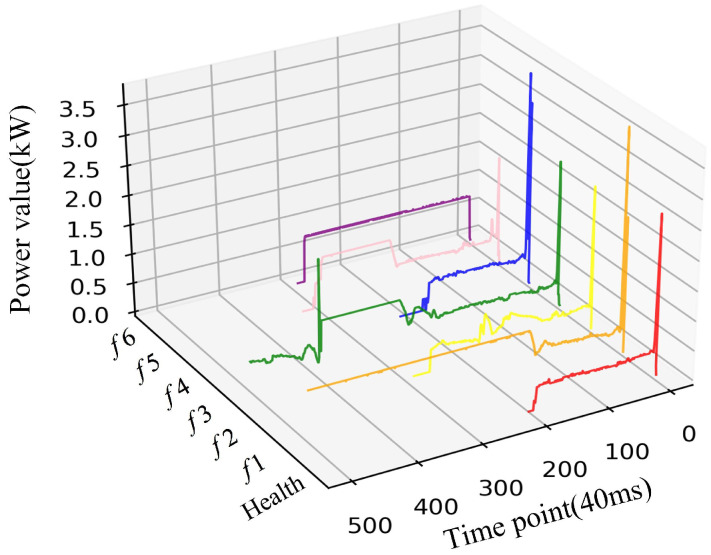
Turnout failure power curve.

Trains rely heavily on turnouts during high-speed operation, and the health of turnouts affects whether trains will fail when passing through turnouts to a certain extent. Due to long-term exposure to the outdoor environment, the influence of the surrounding environment (weather, etc.), as well as the aging of the equipment and the complexity of the electromechanical structure, affects whether the turnout is prone to failure. The power curve trend of RTS will indicate abnormalities then the fault occurs. For example, if there is a foreign object between the tip rail and the base rail, it will cause failure. The manifestation of the abovementioned situation is that the power value stabilizes at a high level during the conversion phase and holds. This failure mode is marked as *f*1. There are abnormal fluctuations in the transition phase when there is poor contact between the carbon brush of the switch machine and the steering gear, which is marked as *f*2. In addition, based on previous research [16,21], manual screens, and markers by experts, six failure modes in our historical data set were summarized. Figure 2 shows one health and six fault power curves in our dataset, respectively labeled Health, *f*1, *f*2, *f*3, *f*4, *f*5, and *f*6. Table 1 describes in detail six kinds of fault phenomena and causes of turnouts. In the past, manually comparing the collected power curve with the health template is the main fault-diagnosis method. Therefore, the accuracy of identification mainly depends on the work experience of relevant personnel. The method is not only time consuming and labor consuming but also obviously no longer applicable today when the data volume is increasing.

However, due to the working characteristics of the turnout itself, the collected data is typical imbalanced data. Railway transportation is carried out every day; a line has many stations, a station has many turnouts, and the railway department can detect a large amount of data every day through the MMS. In the monitoring statistics of a railway station in China over three months, a total of more than 100,000 pieces of data for turnouts signal were collected, including 821 marked known fault data (data category as shown in Table 1). However, there are more than 100,000 pieces of health data. Because health data accounts for the vast majority, if we perform fault detection on imbalanced data, the model is more inclined to learn health data features. However, the model is not sensitive enough to abnormal data and cannot fully learn the essential characteristics of fault data, which affects the classification and recognition performance of the model. Therefore, solving the problem of uneven data is of great significance to improve the performance of the diagnostic model.

## 3. Method

**Figure 3:**
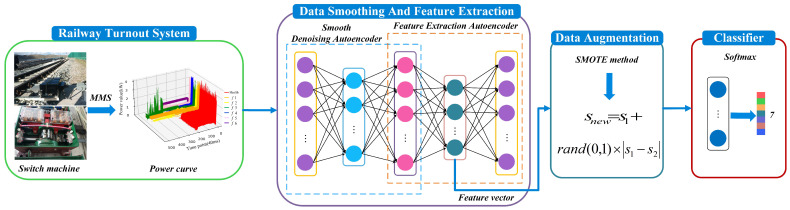
The framework of the proposed fault diagnosis method.

Figure 3 shows the framework of the proposed fault-diagnosis method, which mainly includes three modules. (i) First is the AE-based smooth denoising and feature extraction. Here, the turnout power curve dataset is smoothed and denoised by the sliding mean as the target output result of the smooth denoising autoencoder (SDAE), and train SDAE to reduce the effect of noise. Two fully connected layers are stacked after the SDAE, and its output is used as the input of the feature extraction autoencoder (FAE), and the latent feature vector of the data is obtained through the representation of its hidden layer as the result of latent feature extraction. (ii) Second is data augmentation. By synthesizing minority oversampling technology, data balance is performed on the potential feature dataset of faults to solve the problem of data imbalance. (iii) Third is fault diagnosis based on Softmax classification. The Softmax classifier has the characteristics of small training time and high accuracy in classification. For the potential feature data of the obtained balanced dataset, using the Softmax classifier can effectively shorten the training time and have high classification accuracy.

### 3.1. AE-Based Smooth Denoising and Feature Extraction

Autoencoder (AE), which is based on the backpropagation algorithm and optimization method, and which utilizes an input layer vector *x* to direct a neural network to learn a mapping relationship (so as to obtain a reconstructed output $\hat{x}$), is an unsupervised learning model consisting of an encoder and a decoder. The function of the encoder is to encode the high-dimensional input *x* into the low-dimensional hidden variable *h*, forcing the neural network to learn the potential characteristics of the input signal, and its encoding formula is shown in

(1) $$h=\sigma (w_{1}x+b_{1}),$$

where w1 and b1 are the weight matrix and bias vector of the encoder, respectively, σ () is activation function usually the Relu function replaces the s-type function as the hidden layer activation function. In addition, the decoder reconstructs *x* from *h* by taking the output vector of the encoder as the input vector, and its decoding formula is shown in

(2) $$\hat{x}=g\left(h\right)=\sigma \left(w_{2}x+b_{2}\right),$$

where w2 and b2 are the weight matrix and bias vector of the decoder, respectively, σ () is the decoder layer activation function, and $\hat{x}$ is the reconstruction vector of *x*. The reconstruction error of AE is minimized to ensure that the reconstructed signal can recover the original input signal as much as possible, and the optimization objective function of the algorithm is as follows,

(3) $$Minimizeloss=dist(x,\hat{x}),$$

where dist() is the distance measurement function of the two, usually MSE, and its formula is shown in

(4) $$dist\left(x,\hat{x}\right)=||x-\hat{x}{\left|\right|}^{2}=\frac{1}{n}\sum_{i=1}^{m}\left|\right|x_{i}-{\hat{x}}_{i}{\left|\right|}^{2}.$$

**Figure 4:**
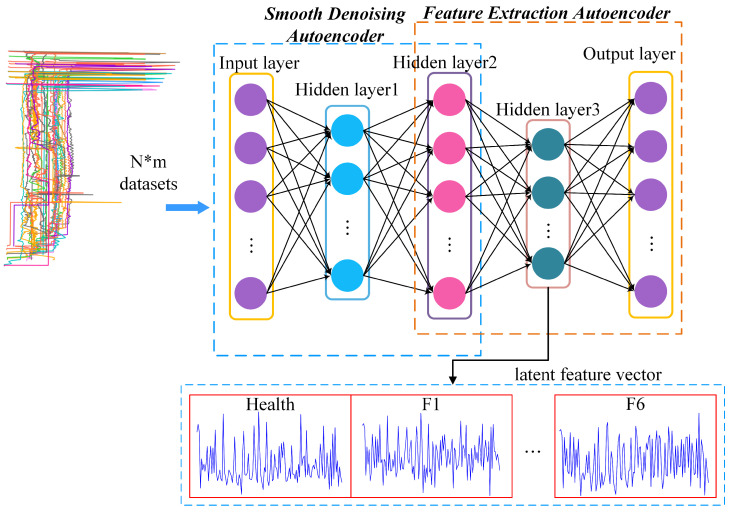
Frame diagram of the autoencoder combined with smooth denoising and feature extraction.

Because it deals with complex problems, AE with only one hidden layer has a poor effect, and the more hidden layers there are, the more abstract is the extracted feature information. Stacked autoencoder (SAE) is a deep neural network model composed of multiple layers of sparse autoencoders, and the output of the hidden layer of the previous layer of autoencoders is used as the input of the latter layer of autoencoders. Due to its unsupervised latent feature learning ability, SAE can effectively express a large number of unlabeled data features, which provides a new solution for feature extraction of turnout. The main idea behind this method is to learn a function through an unsupervised learning algorithm that makes the output value close to the input value to the greatest extent. This paper mainly realizes smooth denoising and feature extraction of data based on SAE. The method framework is shown in Figure 4. This model can not only smooth the signal data to facilitate feature recognition, but also use the ability of AE to extract deep, potential, and comprehensive features.

#### 3.1.1. Smooth Denoising

The switch machine is driven by electric energy, but due to the physical characteristics, the noise will be generated during the electric conduction process, and the monitored power curve will have normal fluctuations. As shown in Figure 1, the health power curve of the turnout can be observed in the second and third stage fluctuations. However, due to the mechanical and electrical equipment damaged, the contact is poor, and the surface of the friction belt is not smooth, etc. There will also be fluctuations in the power curve of the faulty turnout, but the amplitude of such fluctuations will be significantly smaller than the fluctuation noise of the healthy power. However, the influence of current or power noise fluctuation will infect the accuracy of fault diagnosis. Therefore, it is of great significance to smooth and denoise the signal in fault diagnosis.

The method we adopted is to carry out moving average denoising in the second and third stages of the power with obvious noise fluctuation, and the segmentation method of stages is referred to literature [40]. After segmentation, the second stage is denoised by Formula (5), and the third stage is denoised by Formula (6):

(5) $$y\left(n\right)=\frac{1}{3}\left(x\left(n-1\right)+x\left(n\right)+x\left(n+1\right)\right)$$

(6) $$y\left(n\right)=\frac{1}{2}\left(x\left(n\right)+x\left(n+1\right)\right).$$

**Figure 5:**
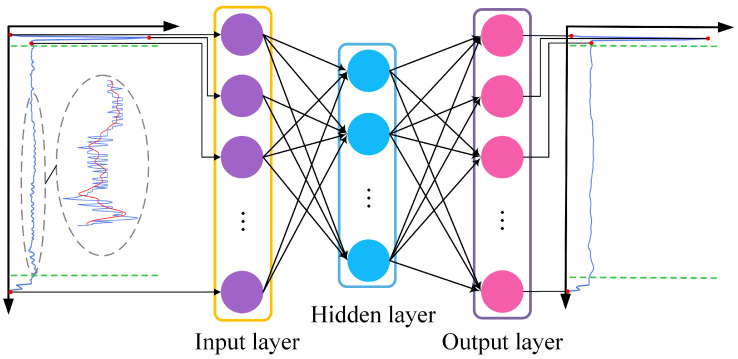
Moving average smoothing denoising effect.

Denoising on the health data can smooth the second and third stages of the health action curve. Because the fluctuation of the fault action curve is large, the effect of using the sliding mean denoising is not obvious, and the fault characteristics are preserved. This method can better distinguish healthy action curves from faulty action curves, and we adopt this method for the construction and selection of high-quality datasets. Figure 5 shows the effect of denoising the healthy power data with sliding mean smoothing.

#### 3.1.2. Feature Extraction

AEs can obtain the internal feature expression of the dataset through supervised learning, extract useful information, and achieve the purpose of feature extraction. When the output value close to the input value to the greatest extent the hidden layer of the AEs can characterize the latent features of the input vector. However, the AE does not mean that the higher the similarity between the output data and the input data, the better. If the similarity is too high, overfitting may occur. What we hope is that the AE can encode the same type of data, that is, it requires a strong generalization ability. In order to achieve this goal, we add regularization to the encoder in the experiment, so that the AE can learn sparse features.

In this paper, we use a stacked self-encoder (SAE) to combine smooth denoising and feature extraction, specifically, two layers of fully connected networks are stacked behind the smooth denoising AEs after pre-training. One layer is used as the hidden layer of FAE, the other layer is used as the output layer, and the output of DAE is used as the output layer of FAE.

### 3.2. Data Augmentation

Before fault diagnosis, the data imbalance problem needs to be addressed. Because the proportion of faulty samples in all data samples is very small, it may affect the training of the classification model, which is more inclined to learn the characteristics of healthy samples and ignore faulty samples. Therefore, imbalanced data often leads to the failure of some machine learning models. In this paper, the SMOTE technique, an improved method, is applied to deal with the imbalanced data. Unlike traditional oversampling methods based on sample replication, SMOTE generates new samples for new minority class samples by taking partial minority class samples and adding random numbers to them, and the process is as follows.

(1) Select each sample $s_{i}$ in turn from the minority class samples as the root sample for synthesizing new samples.

(2) According to the upsampling ration, find its *k* nearest neighbors (*k* is generally an odd number, such as k=5) from the same class of $s_{i}$, and randomly select a sample as an auxiliary sample for synthesizing a new sample, and repeat *n* times;

(3) Linear interpolation is performed between the sample $x_{i}$ and each auxiliary sample by Formula (7).

(4) *n* synthetic samples are generated. Among them, $s_{i}$ is the *i*th sample in the minority class, $s_{ij}$ is the *j*th nearest neighbor sample of the sample $s_{i}$, j=1,2,…,k; γ is a random number between [0, 1]; $s_{new}$ represents in $s_{i}$ A new sample synthesized with $s_{ij}$ is

(7) $$s_{new}=s_{i}+\gamma \times \left|s_{i}-s_{ij}\right|.$$

### 3.3. Fault Diagnosis Based on Softmax Classification Models

Softmax regression is an extension of logistic regression (LR), which is different from logistic regression classification, which can only take two class labels. It is widely used in the supervised learning part of depth learning research as a classifier. It provides more possibilities for class labels and is suitable for multiclassification problems. The principle of the Softmax classifier is relatively simple, and it is a probability calculation process. After the input data is trained by the Softmax classifier, the parameter matrix θ can be obtained, the θ is multiplied by the feature vector, and the probability value of each category is output. Among them, the category corresponding to the maximum value is the judgment category. The Softmax classifier maps input vectors from an N-dimensional space to categories, and the results are given in the form of probabilities with the following Formula (8).

Input data *x* = {($x_{1}$, $y_{1}$), ($x_{2}$, $y_{2}$), …, ($x_{m}$, $y_{m}$)} have *k* types, and the Softmax regression mainly estimates the probability that the input data [formula] belongs to each class, as shown in the Formula (8):

(8) $$h_{\theta }\left(x_{i}\right)=\left[\begin{array}{c} p(y_{i}=1|x_{i};\theta )\\ p(y_{i}=2|x_{i};\theta )\\ \vdots \\ p(y_{i}=3|x_{i};\theta ) \end{array}\right]=\frac{1}{\sum {}_{j=1}^{k}e^{\theta_{j}^{T}x_{i}}}\left[\begin{array}{c} e^{\theta_{1}^{T}x_{i}}\\ e^{\theta_{2}^{T}x_{i}}\\ \vdots \\ e^{\theta_{k}^{T}x_{i}}, \end{array}\right]$$

where $\theta_{1},\theta_{2},...,\theta_{k}\in \theta$ is the parameter of the model, multiplied by $\frac{1}{\sum {}_{j=1}^{k}e^{\theta_{j}^{T}x_{i}}}$, so that the probability is at [0, 1] and the sum of the probabilities is *l*, the probability that Softmax regression assigns the input data $x_{i}$ to the category *j* is

(9) $$p\left(y_{i}=1|x_{i};\theta \right)=\frac{e^{\theta_{j}^{T}x_{i}}}{\sum {}_{l=1}^{k}e^{\theta_{l}^{T}x_{i}}}.$$

The parameter matrix θ of Softmax regression can be written as

(10) $$\theta =\left[\begin{array}{c} \theta_{1}^{T}\\ \theta_{2}^{T}\\ \vdots \\ \theta_{k}^{T} \end{array}\right].$$

The cost function of Softmax regression is shown in Formula (11):

(11) $$L\left(\theta \right)=-\frac{1}{m}\left[\begin{array}{c} \sum {}_{i=1}^{m}\sum {}_{j=1}^{k}\left\{y_{i}=j\right\}\log\frac{e^{\theta_{j}^{T}x_{i}}}{\sum {}_{l=1}^{k}e_{l}^{T}x_{i}} \end{array}\right..$$

## 4. Experiment

**Figure 6:**
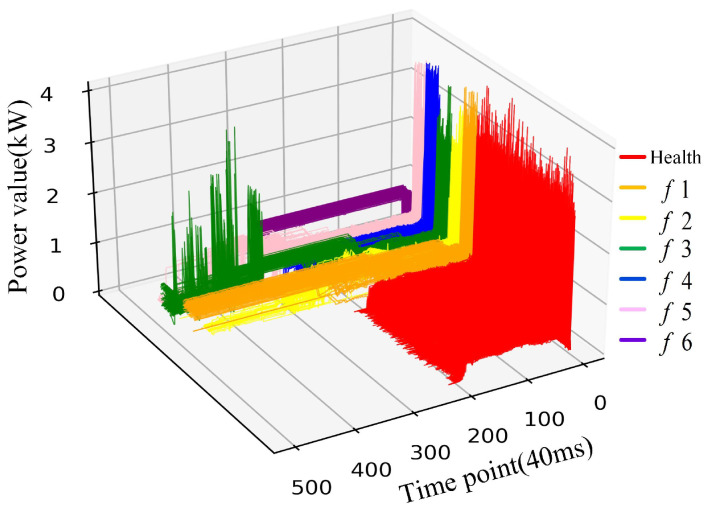
The power curve dataset selected for the experiment.

To verify the effectiveness and advanced nature of the proposed method, we use Tensorflow 1.15.0 framework in Python 3.7 as the computational algorithm for the experiment, which was conducted by using three months of a turnout power signal collected at a railway station in China, where the types of the switch were ZD6. Figure 6 shows a curved three-dimensional graph of the dataset, including 1000 health examples and 780 fault examples (130 faults of each type). The health data and fault data are majority-class and minority-classes, respectively, and the imbalanced degree of them is η = 7.69.

### 4.1. Evaluation Metrics

Evaluation metrics matter a great deal to the model of RTS fault diagnoses, such as accuracy, precision, recall, and F1-score. In Table 2, these metrics are mainly used in the evaluation of the results in this paper, which can give us a macro-level understanding of the model effect. True positive (*TP*) and true negative (*TN*) refer to the number of correctly classified positive and negative instances, respectively. The number of negative cases and positive samples wrongly categorized are defined as false positive (*FP*) and false negative (*FN*). In general, the diagnostic model will perform better with a higher evaluation index value.

### 4.2. Experimental Parameter Settings and Result

According to the literature [28], the dataset was preprocessed by normalization, and the measure of adding zero for the data lengths less than 500, and cropping was used for those greater than 500. In the SD–FAE processed stage, 30 pieces of data each for health and failure are selected, and the model is trained by using the method in Section 3.1.1. The SD–FAE model consists of an input layer, three hidden layers, and an output layer. The size of the input layer and the output is 500, and the hidden layer is 200, 500, and 128 by experiments, respectively. Then, SDAE was trained, which includes the first three layers of SD–FAE, and the output is the second hidden layer. For the training, Adam is selected as the optimizer, and the learning rate, epoch, and batch size are set to 0.001, 1000, and 32, respectively.

**Figure 7:**
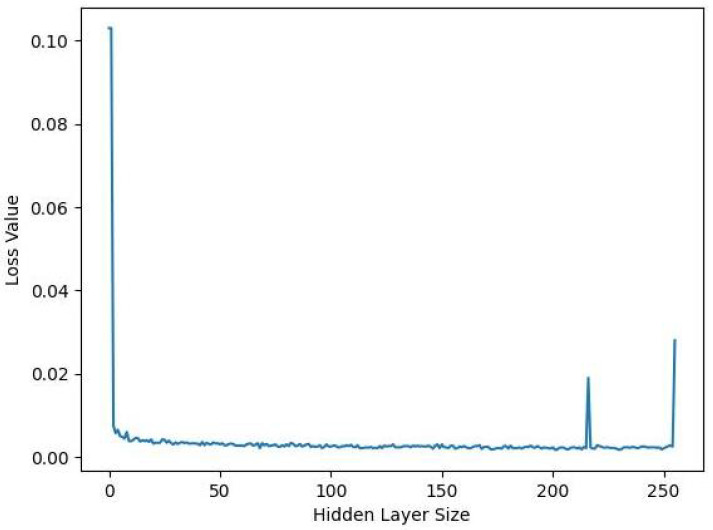
Loss changes with the hidden layer size changes.

SD–FAE is to add two layers of the network after SDAE, as the hidden layer and the output layer of FAE, respectively. By training the SD–FAE, we split off the final output layer to obtain the feature vector of the input signal. To determine a suitable number of hidden layers, we used the iterative method, using the parameter settings of the training SDAE, and obtained the loss plot of SD–FAE, as shown in Figure 7. We can see that the loss remains stable afterward, so it has little effect on the model afterward, and we chose 128 as the number of cells from the more intermediate data in the hidden layer; both the number of features is 128.

**Figure 8:**
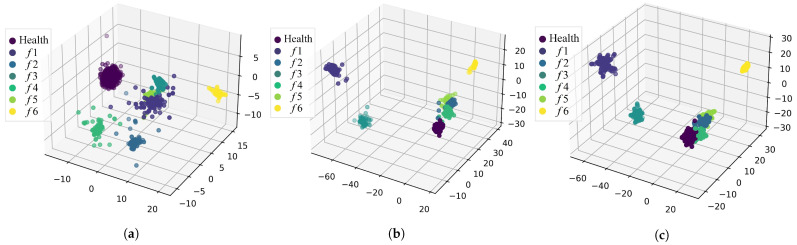
3D Vvsualization of LDA. (**a**) Unprocessed data. (**b**) Feature data obtained from SD-FAE. (**c**) Feature data processed by SMOTE.

To verify the feature extraction effectiveness of SD–FAE, LDA were utilized to reduce the dimension visualization for the raw data. Feature data obtained from SD-FAE, and feature data after data augmentation of RTS are shown in Figure 8. Compared with the dimensionality reduction visualization of the original dataset, the data categories after feature extraction can be observed more clearly in the visualization. In Figure 8a, we can see that except for fault 6, the sense of boundary between the data of each category is not obvious, and there is a crossover between the categories. We are able to see that in addition to *f*6, *f*1 and *f*3 are also able to clearly separate out the boundaries, and the clustering effect of other categories is more obvious in Figure 8b. In Figure 8a, with the exception of *f*6, which forms a cluster separately, there is no clear distinction between the other categories. In fact, the trend of the *f*6 curve is obviously different from that of other categories. It does not form a downward trend and will be stable at a value. Three well-separated groups of clusters are apparent in LDA plots, corresponding to the *f*1, *f*3, and *f*6 categories. Then, our proposed model is verified to be able to find the hidden information.

The data after data augmentation should belong to the same class, so the same LDA visualization analysis of the feature dataset after augmentation by using SMOTE is performed in this paper. In Figure 8c, it can be seen that SMOTE data augmentation can effectively enhance the increase of data in the local range of samples in the fault category, and the naked eye can more clearly distinguish the boundaries, which indicates the effect of the SMOTE method for feature augmentation of feature data. The distribution of data after SMOTE data augmentation remains basically the same. The added data are distributed around the area of the same category and were not distributed by the enhanced SMOTE data, which remains basically the same.

For the fault diagnosis from our imbalanced data, it is necessary to further use the evaluation criterion of the Section 4.1 to analyze the diagnostic results. Based on the same dataset, the proposed method is compared with four commonly used fault diagnosis models (SVM, KNN, XGBoost, LSTM) and the models in a two-year period (SVM-based [40], 2DCNN-based [24], and 1DCNN-based [41]). Subsequently, the average classification accuracy, precision, recall, and f1-score of each health condition in Health–*f*6 is shown in Table 3. It can be seen that the evaluation criterion of our method (SD–FAE+SMOTE+Softmax) is over 99.10%. Compared to the four experiments on the left, the four pairs on the right all performed feature extraction on the data, and the experimental results demonstrated better results in all four evaluation criteria. Obviously, it is exceedingly helpful to improve diagnostic accuracy with special data preprocessing and feature extraction for data in RTS fault diagnosis. In addition, it can be seen that the evaluation criterion of our method (SD–FAE+SMOTE+Softmax) is over 99.10% and is superior to other models. Although the literature [40] uses fixed formulas to extract features from segmented signals, this method is limited by the meaning of the formula. Based on deep learning, the 2DCNN- based method [24] has a superior performance in pattern recognition. However, it does not pay attention to the noise. The method [41] utilized improved the wavelet to eliminate noise and extract time–frequency features. However, it may cause information loss in the reconstruction process and affect classification accuracy. In contrast, the proposed SD–FAE based method can accurately realize pattern recognition. Therefore, it is more effective and practical in the fault diagnosis of RTS.

With the same experimental steps and the same evaluation indicators, unsmoothed data is used for training the SD–FAE model, and the results are in Table 3. Compared with the addition of the smooth denoising method, the effect of unsmoothed data on the SD–FAE model is not as good as the proposed method. The main reason is that the SD–FAE model trained in the above way has no function of smooth denoising, which also shows that effective denoising can help with fault diagnosis of RTS.

**Figure 9:**
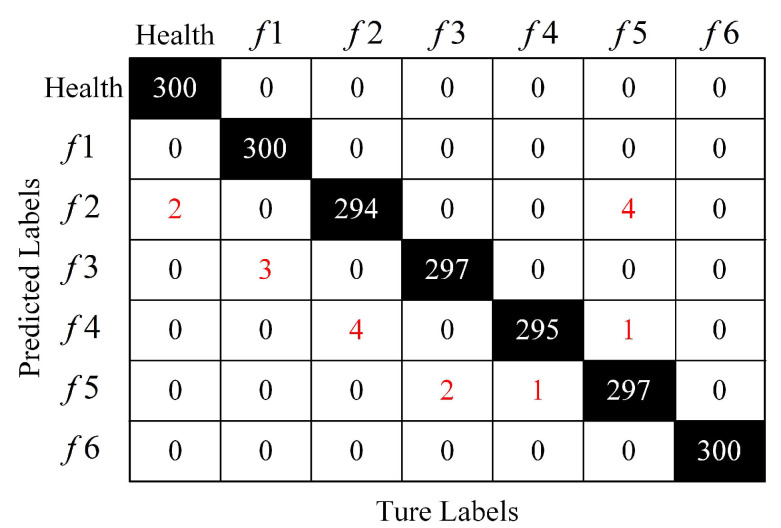
Confusion matrix of proposed method.

The confusion matrix, as a standard format of accuracy evaluation in unsupervised learning, is used to evaluate the fault diagnosis results of RTS, and the method of 10 random samplings’ repeated tests is used for verification, as shown in Figure 9. The elements on and outside the diagonal represent the number of samples correctly predicted (i.e., predicted condition = actual condition) and incorrectly predicted, respectively. Only *f*2 has a missed diagnosis, whereas *f*3 and *f*4 have a missed diagnosis. Noticeably, there is no missed diagnosis or misdiagnosis in the Health, *f*1, and *f*6 categories. There are more than 99% classification effects in the recognition of each class, which shows the effectiveness, accuracy, and stability of our method.

## 5. Conclusions

In this paper, a fault-diagnosis method based on SD–FAE, SMOTE, and Softmax is proposed to diagnose the turnout power signal in the RTS. First, an improved autoencoder that incorporates smooth denoising and feature extraction is suggested. A high-quality dataset is constructed by using the smooth denoising method as the target output of the SDAE. Secondly, due to the unsupervised adaptive potential feature extraction ability of the autoencoder, two full connection layers are stacked after the SDAE, which is used to get the deep characteristics of the turnout signal. Thirdly, in terms of data preprocessing, to solve the problem of data imbalance, the SMOTE algorithm is used to expand six fault categories. Finally, the health state is identified by the Softmax regression model that is trained with the balanced characteristics data, which improves the diagnosis precision and generalization ability. The experiment uses the turnout power signal data of different track sections of a railway station in China to evaluate, and the results show that this method has a performance superior to the existing methods.

Currently, the proposed method only relies on a single turnout power signal, which has not been verified in double turnouts and multiple turnouts in the RTS. In fact, double turnouts and multiple turnouts are more complex and harder to diagnose; they are also widely used in the railway field. Moreover, in addition to a single failure, it is equivocal that many key equipment failures are coupling failures between multiple failure modes. Therefore, future research directions of this paper may include studying a more intelligent method for the fault of double turnouts and multiple turnouts and coupling failures between multiple failure modes.

## Figures and Tables

**Table 1:** Six kinds of fault phenomena and causes of turnouts.

Category	Description	Cause of Failure
*f*1	During the conversion phase, the power value stabilizes at a high level and holds.	There are foreign matters between switch rail and stock rail.
*f*2	There are abnormal fluctuations in the conversion phase.	Poor contact between the carbon brush of the switch machine and the steering gear. The steering gear has broken grids or poor surface. The friction belt surface is not smooth.
*f*3	There are abnormal fluctuations in the locking phase.	There are damaged diode devices in a cable box.
*f*4	There are two peaks in the uplocking phase.	There are poor automatic switching equipment contacts.
*f*5	The power value falls back after rising for a while in the locking phase.	Turnout adjustment is too tight, sliding bed plate is too dirty, sliding bed plate is out of oil, turnout hanger plate, stock rail traverse.
*f*6	The power curve does not form a downward trend and will be stable at a value.	The stator and rotor of the switch machine have mixed lines.

**Table 2:** Evaluation metrics methods in the RTS fault diagnoses.

Metrics	Formula	Meaning [33]
Accuracy	Accuracy = $\frac{TP+TN}{TP+FN+FP+TN}$	An overall correct prediction rate on the test set.
Precision	Precision = $\frac{TP}{TP+FP}$	A ratio of correctly predicted positive samples to the all predicted positive samples.
Recall	Recall = $\frac{TP}{TP+FN}$	A ratio of the number of samples correctly classified as positive in the test sample to the actual number of positive samples.
F1-score	F1-score = $\frac{2*Precision*Recall}{Precision+Recall}$	Weighted harmonic average between Precision and Recall.

**Table 3:** Comparison of evaluation index results between ours and the other seven methods.

Metrics	Method								
	SVM	KNN	XGBoost	LSTM	SVM [40]	2DCNN [24]	EBTW + 1DCNN [41]	Without Smoothing	Ours
Accuracy (%)	96.80	93.19	94.29	94.46	98.50	98.03	98.57	97.52	99.13
Precision (%)	97.92	95.70	98.31	94.96	99.01	98.12	98.73	97.57	99.20
Recall (%)	98.19	93.19	95.28	94.70	98.56	97.56	98.31	97.52	99.14
F1-score (%)	98.00	94.21	96.67	94.35	98.00	98.03	98.67	98.55	99.17

## Data Availability

The data presented in this study are available on request from the corresponding author.

## Abbreviations

The following abbreviations are used in this manuscript:

RTS	Railway turnout system
MMS	Microcomputer monitoring system
SVM	support vector machine
KNN	K-Nearest Neighbor
CNN	Convolutional neural network
PCA	principal component analysis
SMOTE	Synthetic minority over-sampling technique
AE	Autoencoder
SDAE	Smooth denoising autoencoder
FAE	Feature extraction autoencoder
LDA	Linear discriminant analysis
